# Construction of the Red Swamp Crayfish (*Procambarus clarkii*) Family Selection Population and Whole Genome Sequencing to Screen *WIPFI* Candidate Genes Related to Growth

**DOI:** 10.3390/genes16020174

**Published:** 2025-01-31

**Authors:** Xing Tian, Xiudan Yuan, Zhigang He, Weiguo Li, Jinlong Li, Yong He, Shiming Deng, Jiarong Guo, Miaoquan Fang, Dongwu Wang

**Affiliations:** 1 Hunan Provincial Key Laboratory of Nutrition and Quality Control of Aquatic Animals, Hunan Fisheries Science Institute, Changsha 410153, China; tianx2323@163.com (X.T.); yuanxd2024@163.com (X.Y.); oceanhzg@163.com (Z.H.); wsljl2007@163.com (J.L.); heyong19891989@outlook.com (Y.H.); dsmjgm@126.com (S.D.);; 2 Huazhi Biotechnology Co., Ltd., 618 Heping Road, Changsha 410153, China; weiguo.li@higentec.com

**Keywords:** *Procambarus clarkii*, family selection, GWAS, *WIPF1*

## Abstract

**Background/Objectives:** *Procambarus clarkii* is an important freshwater aquaculture species in China which has the characteristics of rich nutrition and delicious taste. However, the expansion of aquaculture scale, germplasm degradation, and other problems that have become increasingly prominent seriously restrict the sustainable development of the crayfish industry. Genetic improvement is an urgent need for the crayfish aquaculture industry, and selective breeding is an important way to improve the crayfish varieties. **Methods:** We established full-sibling family populations of the red swamp crayfish and performed whole-genome resequencing of the F3 family-selected red swamp crayfish population and wild red swamp crayfish populations from four regions of Hunan Province (Nanx, Mil, Caish, and Wangc). **Results:** The results showed that there was a clear separation between the wild population and the family population, and the decline rate was slightly faster in the wild population than that of the family breeding population. There was local gene flow between family populations, as well as gene flow between Mil, Caish, and families. In addition, 52 SNP loci related to body weight traits were identified by genome-wide association analysis, and the candidate gene *WIPF1* related to growth was screened out. **Conclusions:** We established a line selection population of red swamp crayfish and obtained more stable candidate lines. In addition, this study identified *Wiskott–Aldrich syndrome protein-interacting protein family member 1 (WIPF1)* as a candidate gene related to body weight for the first time. The results provide a theoretical basis for exploring the growth mechanism of *P. clarkii* and carrying out in-depth genetic improvement.

## 1. Introduction

The red swamp crayfish (*P. clarkii*), native to North America, is one of the most important freshwater species in Chinese aquaculture and has significant economic value [1,2,3]. In recent years, the production of crayfish in China has surged. In 2023, the production of crayfish exceeded 3.16 million tons, accounting for 9.26% of the national freshwater aquaculture production. It has become the fourth largest freshwater aquaculture species after grass carp (*Ctenopharyngodon idella*), silver carp (*Hypophthalmichthys molitrix*), and bighead carp (*Aristichthys nobilis*) [4]. At present, the culture modes of red swamp crayfish are various, such as paddy fields, ponds, culture buckets, and rice–shrimp symbiosis, of which the rice–shrimp aquaculture model has attracted much attention because of its high economic benefits [1,5,6,7]. However, as crayfish populations continue to increase, the lack of natural predators is causing their numbers to rise. In addition, due to its meat quality, its nutritiousness, and other characteristics of high commercial value, “catching big and leaving small” fishing methods have resulted in reverse selection, which in turn led to the degradation of germplasm resources, individual miniaturization, and the frequent occurrence of diseases and other issues, which have become a key bottleneck factor restricting the sustainable development of crayfish [8,9,10]. The shortage of excellent germplasm has seriously restricted the healthy development of the industry. Therefore, the breeding of improved varieties needs to be strengthened. Crayfish exhibit a high level of natural genetic variation in both farmed and wild populations, and it is possible to breed crayfish for superior traits using genetic and breeding techniques. Methods such as selective breeding and gene editing will play an important role in supporting sustainable increases in aquaculture production [11,12,13,14]. Studies have shown that large-scale lineage selection is a core technology that can support the development of better quality in the vannamei shrimp breeding industry, and, based on the lineage selection breeding model, an open-nucleus breeding system (ONBS) for vannamei shrimp has been designed using computer simulation to accelerate the process of the selective breeding of vannamei shrimp [11]. Therefore, directed pairing and selection within family lines can provide an effective method for genetic improvement. However, limited genetic and genomic resources have resulted in minimal improvement in their breeding programs [15,16].

High-quality genomes are important not only for understanding the genetic basis of biologically and economically important traits but also for the genetic improvement of aquaculture species [17]. Currently, the utilization of crustacean genomics lags far behind that of other animals, and few studies have been reported on the genomic information of crayfish [17]. Yi et al. assessed the population structure pattern and dispersal history of crayfish in China using 2b—RAD technology [18]. Xu et al. reported that the chromosome-level reference genome of red swamp crayfish provides a useful resource for genetic improvement in red swamp crayfish breeding selection programs [19]. Whole genome sequencing is a method of sequencing the whole genome of an individual with a known genome sequence and analyzing the differences at the individual or population level [20,21]. Whole genome sequencing not only provides a genetic basis for the determination and evolution of phenotypic traits, but also provides a useful resource for the genetic improvement of aquaculture species [19,22]. For example, whole genome resequencing was used to reveal recently selected traits in five species of largemouth bass (*Micropterus salmoides*) and predict candidate genes related to growth, early development, and immunity, providing a theoretical basis for future breeding of largemouth bass [23]. For specific species, the large number of SNP markers generated by genome sequencing lays the foundation for the application of genomic selection. Genome-wide association studies (GWAS) are used to analyze the association between genotypes and phenotypes by detecting differences in allele frequencies of single nucleotide polymorphisms (SNPs) among individuals [24,25]. Scallop (*Patinopecten yessoensis*) growth candidate genes are identified using genome-wide association analysis. Genomic regions associated with viral disease resistance were identified in Atlantic salmon (*Salmo salar*) [26,27]. Xiao et al. used genome-wide association analysis (GWAS) to associate a genomic region on chromosome 15 with coat membrane markers in abalone (*Haliotis rubra*) [28]. With the public availability of the whole genome map of crayfish, more molecular-assisted breeding tools are available for new variety creation.

The red swamp crayfish is suitable for large scale farming in China, but individual miniaturization, high morbidity, and other germplasm degradation problems are becoming increasingly prominent, which seriously restrict the healthy and sustainable development of the crayfish industry. Genetic improvement is an urgent need for the crayfish farming industry. However, there are few reports based on the selective breeding of crayfish and its genetic characterization. Therefore, we established a family line selective breeding population of crayfish in this study and conducted whole genome resequencing with the family line selective populations and the wild populations to evaluate the breeding effect of the family line selection. Further combined with the GWAS analysis, candidate genes regulating growth and other excellent traits were screened to provide theoretical support for the selection and breeding of new varieties of crayfish and to accelerate the rate of the genetic gain of crayfish.

## 2. Materials and Methods

### 2.1. Ethics Statement

All experimental procedures were conducted in accordance with the standards and ethical guidelines established by the Academic Committee of the Hunan Fisheries Science Institute, Changsha, China (Approval Code HFSI20220906, Approval Date 6 September 2022).

### 2.2. Construction of Selective Breeding Lines of the Red Swamp Crayfish

The red swamp crayfish were fished and selected from the East of Dongting Lake for centralized breeding, coming in at about 75 kg, which was an average distribution due to three environmental conditions (pond, factory farming barrel, paddy field) of group cultivation. Then, parental crayfish with complete abdominal limbs, high mobility, and weighing more than 35 g were selected from each group for directional pairing to construct the family lines. Jinshuiwan pond bred 47 groups, No. JS01 to JS47; Fengxiang agriculture factory farming barrel bred 27 groups, No. FX01 to FX27; Hunan Institute of Fisheries Science paddy field bred 20 groups, No. SD01 to SD20. Furthermore, nine of the egg-holding parent crayfish from Jinshuiwan pond were randomly selected for irradiation mutagenesis with UV lamps, a power of 40 W, water depth 20 cm, irradiation time of 2 min, 4 min, and 8 min, using a total of 3 groups with 3 crayfish per group, No. RA02, RA04, and RA08. Intra-household male and female segregated breeding and further intra-household selection of high-quality individuals for directional 1:1 pair breeding for continuous intra-household selective breeding was performed.

### 2.3. Sample and Phenotypic Data Collection

**Figure 1:**
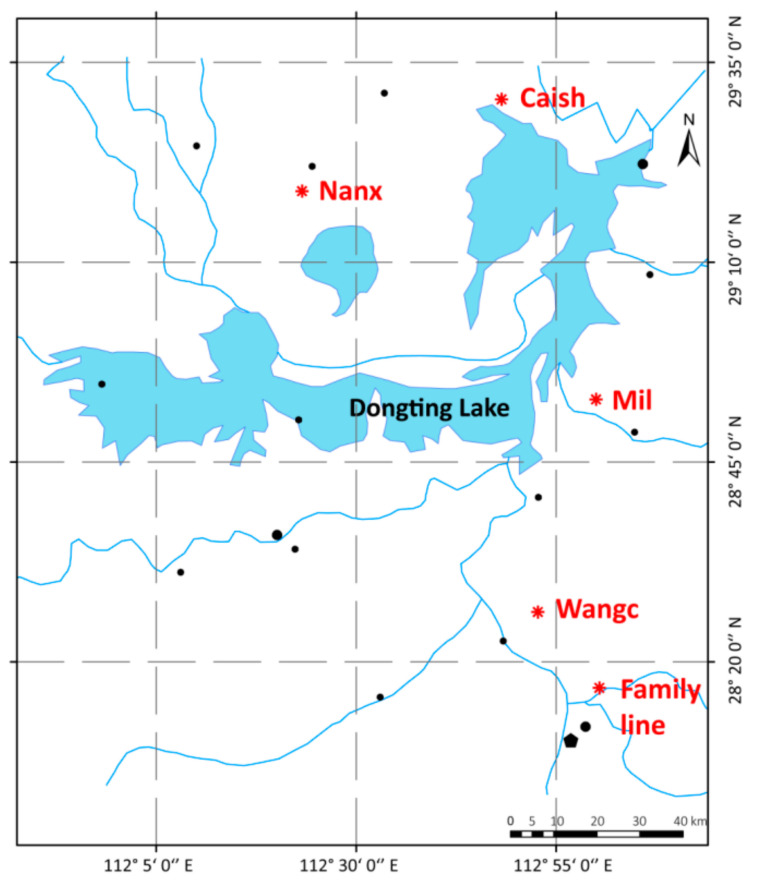
Regional distribution of geographical locations of sample sources. The “❋” in the figure marks the geographic location of the sample source, Nanx stands for Nan County (NanXian, Nanx), Wangc stands for Wangcheng District (WangCheng, Wangc), Mil stands for Miluo City (MiLuo, Mil), and Caish stands for Caisang Lake (CaiSangHu, Caish). In addition, the large dot “⬤” indicates a prefecture-level city seat, the small dot “•” indicates a county-level city seat, and the pentagon “⬟” indicates a provincial capital city.

A total of 20% of the entire sample size of each group was randomly selected as the experimental sample, and the ratio of male to female was 1:1. The samples were composed of family line selection population and wild breeding population. The 117 samples of the family line selection population were derived from the F3 generation of postnatal parents and contained 13 family lines (6 from JS02, 20 from JS03, 6 from JS05, 37 from JS08, 6 from JS19, 6 from JS24, 5 from JS42, 6 from FX05, 4 from FX10, 6 from FX19, 6 from FX21, 6 from FX22, and 3 from RA08). The 80 samples of the wild reproduction population were mainly derived from four different geographic areas, 20 in each region, respectively. The geographic locations of the four regions in Hunan province are shown in Figure 1, including Nan County (NanXian, Nanx), Wangcheng District (WangCheng, Wangc), Miluo City (MiLuo, Mil), and Caisang Lake (CaiSangHu, Caish). Phenotypic indicators, including body length, body weight, abdomen length, abdomen weight, flesh content, X2nd abdomen width, X2nd abdomen length, X5th abdomen width, X5th abdomen length, head length, were obtained by using the crayfish live intelligent detection platform system. Then, the muscles of the inner side of the right cheliped were collected and immediately frozen in liquid nitrogen.

### 2.4. DNA Extraction and Sequencing

For each sample, 0.02 g of cheliped muscle was cut for DNA extraction. The concentration was measured on a NanoDrop 1000 spectrophotometer (Thermo, Waltham, MA, USA), and the DNA with an OD260/280 in the range of 1.8–2.2 was selected and stored at −80 °C for later use. A total of 1.5 µg of genomic DNA was extracted from each sample, which was used as the input material for DNA sample preparation. Sequencing libraries were generated using a Truseq Nano DNA HT sample preparation Kit (Illumina, San Diego, CA, USA) following the manufacturer’s recommendations, and index codes were added to attribute sequences to each sample. Paired-end sequencing libraries with an insert size of approximately 350 bp were sequenced on the Illumina Hiseq PE150 platform. To gain higher-quality paired reads, we removed several types of reads which mainly result from base-calling duplicates and adaptor contamination. This included reads with ≥10% unidentified nucleotides (N), with >10 nt aligned to the adaptor, allowing ≤10% mismatches, with >50% bases having phred quality <5, and putative PCR duplicates generated in the library construction process.

### 2.5. SNP Discovery and Genotyping

The sequencing reads of each sample were quality controlled using the fastp software v0.23.3 with removing low-quality base (more than 50 percent of base with a quality <20, and the number of N base is >5), as well as to remove adapters [29]. Then, the Sentieon DNAseq pipeline (version 202112.06) was used to conduct variant calling, based on following functions: “sentieon bwa mem” function was applied to align reads to the reference genome, “--algo LocusCollector” and “--algo Dedup” functions were applied to remove duplicate reads, “--algo Haplotyper” function was used to call variants in GVCF format, and “--algo GVCFtyper” function was used to conduct joint calling by combining GVCFs across all samples and generated a population VCF [30]. Further, GATK v4.1.7.0 with “VariantFiltration” module was used to detect SNPs using default criteria: “QD < 2.0, FS > 60.0, SOR > 3.0, MQ < 40.0, MQRankSum < −12.5, ReadPosRankSum < −8.0”. The VCFtools (v 0.1.17) software was then used for further quality control as follows: (1) minor allele frequency (MAF) ≥ 0.05; (2) SNPs with missing rate ≤ 0.1; (3) use bi-allelic SNPs. A total of 8,627,048 SNPs that passed the quality filter remained for further analysis.

### 2.6. Population Structure and Genetic Relatedness

Based on 8,627,048 high-quality SNPs, the population genetic diversity of all samples was investigated via a principal components analysis (PCA) conducted in PLINK (v 1.9), which used “--pca” to perform a PCA, based on a distance matrix constructed in PLINK, using “ape”, which is a package written in the R studio (R version 4.3.0), to construct a neighbor-joining tree. The proportion of mixed ancestry was assessed using the ADMIXTURE software v1.3.0 to investigate the data structure in terms of distinct populations, and the number of ancestries (K) to be used from 1 to 9 was evaluated via a cross-validation (CV); a model with minimal CV errors was selected for further analysis.

### 2.7. Linkage Disequilibrium Analysis

Linkage disequilibrium (LD) decay analysis was conducted by PopLDdecay software v3.42 with default parameters to calculate the nonrandom associations of alleles at different loci for 8,627,048 SNPs [31]. The values of r^2^ were calculated using previously reported formulae, and the final LD decay plot based on r^2^ was drawn using a perl script provided by PopLDdecay [32].

### 2.8. Gene Flow and Ancestral Introgression

We used the data set of 226,463 SNPs, which was obtained by the SNPs, with missing rates equal to 0 and SNPs with LD filtered: variant pruning by PLINK with parameters of “indep-pairwise 100 20 0.2”. Then, we used TreeMix v. 1.13, which is based on a maximum-likelihood population graph and identifies pairs of populations with a higher covariance of allelic frequencies than expected under the no migration model to discern between shared ancestral polymorphism and gene flow [33].

### 2.9. Calculation and Analysis of Heritability

Genetic variance was obtained by whole genome sequencing, SNP screening, and genotype filling using beagle. After that, sex was used as a fixed effect and location as a random effect to calculate the heritability calculation of traits in crayfish using Hiblup (https://www.hiblup.com/).

### 2.10. Genome-Wide Association Studies

A total of 5,291,008 SNPs with missing rate ≤ 0.05 were screened for whole genome-wide association studies (GWAS). GWAS was performed using a mixed linear model (MLM) by R software v4.1.3 package rMVP v1.0.0 [34]. The genetic false positive error rate of the population was controlled by *p*-value < 1 × 10^−5^.

### 2.11. RNA Isolation and Real-Time Quantitative PCR

The weight of 1-month-old juvenile shrimps from the same source was weighed, and the same-day-old juvenile shrimps were further divided into two seedling groups with different sizes according to the weight data analysis. The seedlings of the two groups with different sizes were collected, and the total RNA of the whole body of the seedlings was extracted by a Vinozant RNA extraction kit (RC112-01) (Vazyme, Nanjing, China). The quality of total RNA was detected by 1% agarose gel electrophoresis and nucleic acid analyzer, and cDNA was synthesized according to the Vinozant Reverse Transcription Kit (R223-01). The qPCR primers were designed using the Olig7 software 7.60 and were listed in Table S1. The specificity of the primers was verified by RT-PCR and NCBI blast was used to ensure that the sequence similarity was above 99% before applying to qPCR. For qPCR, each 10 μL qPCR reaction mixture contains 1 μL of cDNA, 5 μL of ChamQ Universal SYBR qPCR Master Mix, 0.5 μL of forward and reverse primers with working solution, and 3 μL of DEPC water. Three biological replicates were performed for each sample, each sample was used as a negative control (NTC), and each experiment was repeated three times. The entire reaction was conducted using Roche LightCycler^®^96 real-time quantitative PCR system (Roche, Basel, Switzerland). The results were calculated using the 2^−ΔΔCT^ method.

### 2.12. Statistical Analysis

Statistical analysis was performed using the paired *t*-test to compare two groups. For multiple comparisons, a Bonferroni post hoc test was applied to adjust the *p*-values. Data are expressed as the mean ± standard error of the mean (SEM) from at least three independent experiments. A *p*-value of <0.05 was considered statistically significant.

## 3. Results

### 3.1. Establishment of Family Selection Lines and Analysis of Phenotypic Data

**Figure 2:**
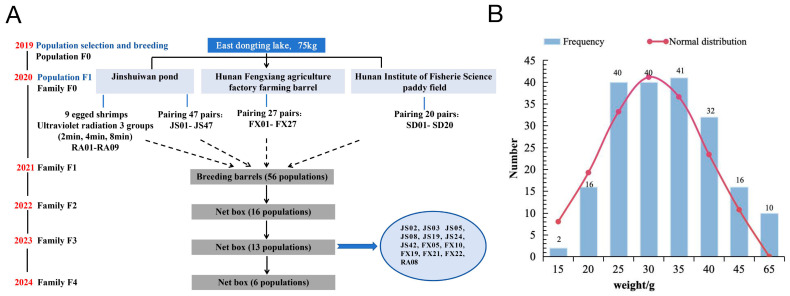
Establishment of a family line of crayfish and results of body weight measurement. (**A**) Flow chart of crayfish lineage establishment. A total of 117 randomly selected samples from the 13 populations in F3; (**B**) Normal distribution histogram of body weight of all samples.

We successfully established full-sibling crayfish lineage selection lines through directed serial breeding (F1–F4), and 13 lineage populations were obtained in F3 (Figure 2A). Further, 117 randomly selected samples from the 13 populations in F3 and 80 randomly selected samples from wild populations originating from four different geographical regions of Hunan Province were used to obtain phenotypic indexes using the crayfish live intelligent detection platform system. A total of 197 samples were found to have body lengths of 80.30 ± 6.76 cm, body weights of 30.74 ± 8.72 g (Figure 2B), abdominal length at 34.61 ± 2.85 cm, abdominal weight at 5.94 ± 0.62 g, flesh content at 10.30 ± 2.41%, X2nd abdomen width at 16.52 ± 1.51 cm, X2nd abdomen length at 6.41 ± 0.55 cm, X5th abdominal width at 14.53 ± 1.41 cm, X5th abdominal length at 6.42 ± 0.61 cm, and head lengths of 34.12 ± 2.88 cm (Table S2).

### 3.2. Sample and Population Evolutionary Genetic Analysis

Genome re-sequencing was performed on 197 samples of red swamp crayfish (117 families and 80 wild red swamp crayfish). A total of 21.7 billion 150 bp double-ended sequences were generated, with a total of 3239.7 GB bases. The average depth of each individual was 6.0×, and the average genome coverage of each individual was 81.0%. After hard filtering with GATK v4.1.7, a total of 32,187,075 SNPs were obtained. After further filtering, 8.63 million high-quality SNPs were retained (Table S3).

**Figure 3:**
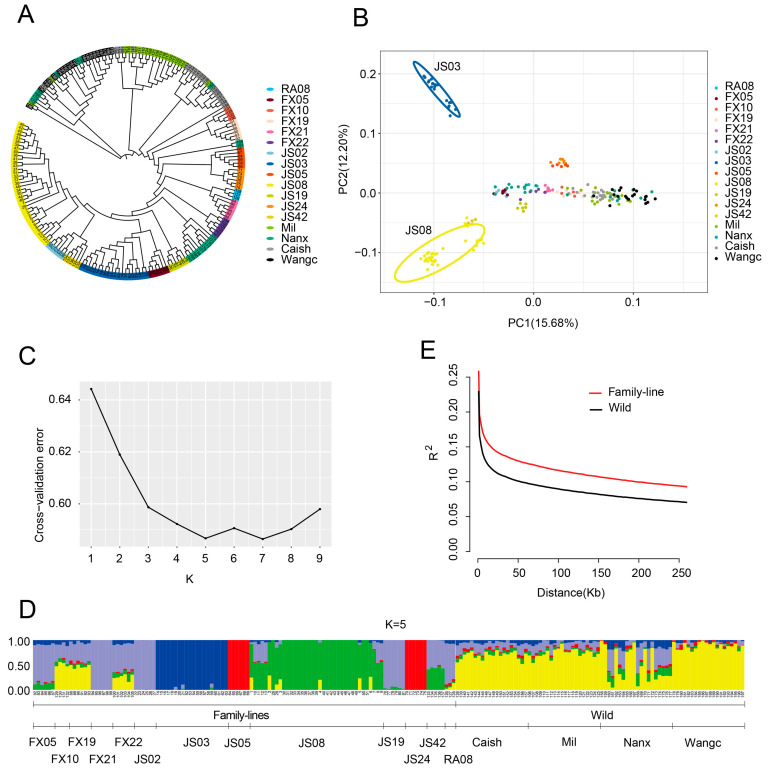
Genetic structure of family lines and wild types crayfish. (**A**) Neighbor-joining (NJ) tree of the 197 individuals constructed using the IBS-distances between individuals, with wild crayfish as an outgroup; (**B**) Plots of principal components 1 and 2 for the 197 individuals; (**C**,**D**) Ancestral composition analysis of 197 individuals; (**C**) Cross-validation plot of K from 1 to 9; (**D**) Stacked barplot for population genetic structure of the crayfish, admixture colour shows the ancestry components ; (**E**) LD linkage disequilibrium analysis of 117 family line samples and 80 wild samples.

In order to explore the genetic background of family lines crayfish, the 8.63 million SNPs were analyzed. We calculated the genetic diversity index of each population, including Expected heterozygosity (He), Observed heterozygosity (Ho), Shannon’s diversity index, minor allele frequency (MAF), Polymorphism information content (PIC), nucleotide diversity, and the inbreeding coefficient. The results showed that these indices were lower than those of the wild population, indicating that the genetic diversity of the family population was higher (Table 1). A population phylogenetic tree was constructed using wild crayfish as an outgroup. The results showed that the family lineages could be divided into separate clusters, such as JS02, JS03, JS08, and JS42, as the genetic distance from the natural population is relatively far (Figure 3A). Principal component analysis (PCA) results showed that some family lines such as JS03, JS08, JS05, JS24, and JS19 were significantly separated from the wild population (Figure 3B). Intentional or unintentional cross-breeding during domestication has been a central process throughout the history of crayfish. To assess the potential impact of these events on the genomes of sample populations, we explored hybridization events and provided additional insights into population structure. As obtained from the ADMIXTURE analysis, K = 5 was the most likely number of genetically differentiated groups (Figure 3C). At K = 5, the ancestry of most family lines appeared to be more homogeneous, reflecting the differentiation of family line generations of crayfish from other crayfish (Figure 3D). The results of linkage disequilibrium (LD) showed that the LD decay of the family was slower than that of the wild population, and JS42, JS03, and JS08 showed slower LD decay than the wild population (Figure 3E and Figure S2). These results suggest that artificial selection leading to variety formation has a greater effect on LD than on nucleotide diversity. The above results of population structure and linkage disequilibrium indicated that there was significant genetic differentiation between family line and wild through multiple generations of directed pair breeding, and the differentiation was more pronounced in JS03 and JS08.

### 3.3. Gene Flow Analysis

**Figure 4:**
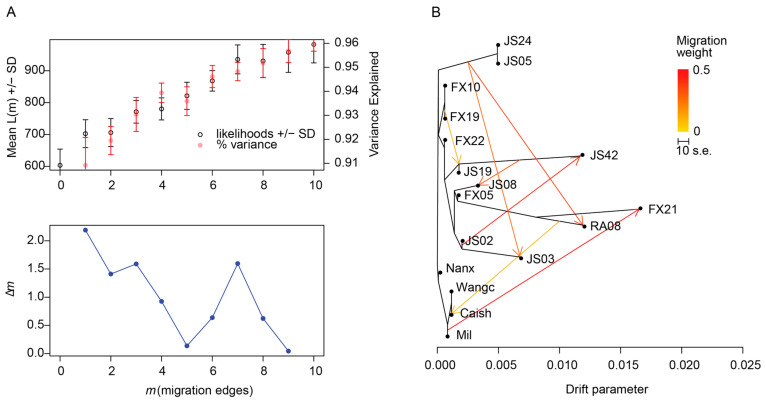
The results of TreeMix analysis. (**A**) Optm fit plots, which evaluated different m results of the TreeMix, showing the best fit (m = 7); (**B**) the likelihood tree of all groups with the direction and degree of gene penetration, the colored arrows show the migration weight from 0 to 0.5.

To further explore the relevance of parental introgression of the original species of the family lineage, we inferred migration events in a maximum likelihood framework by using the wild-type population as an outgroup to infer ancestral states of alleles and to distinguish gene flow from shared ancestral polymorphism. Using OptM to infer the optimal value for m from 0 to 10 simulated migration edges, the highest value for Δm identified the correct number of simulated migration edges for all datasets [35]. According to the OptM results, the TreeMix for m = 7 was most consistent with the statistical rule (Figure 4A). Gene flow analyses showed that there was local gene flow between families, such as between JS02 and JS42, JS42 and JS08, JS24, JS05, and JS03, gene flow between Mil and family FX21, and gene flow between Caish population and family RA08 (Figure 4B).

### 3.4. Analysis of the Heritability of Body Weight and Genome-Wide Association Studies

**Figure 5:**
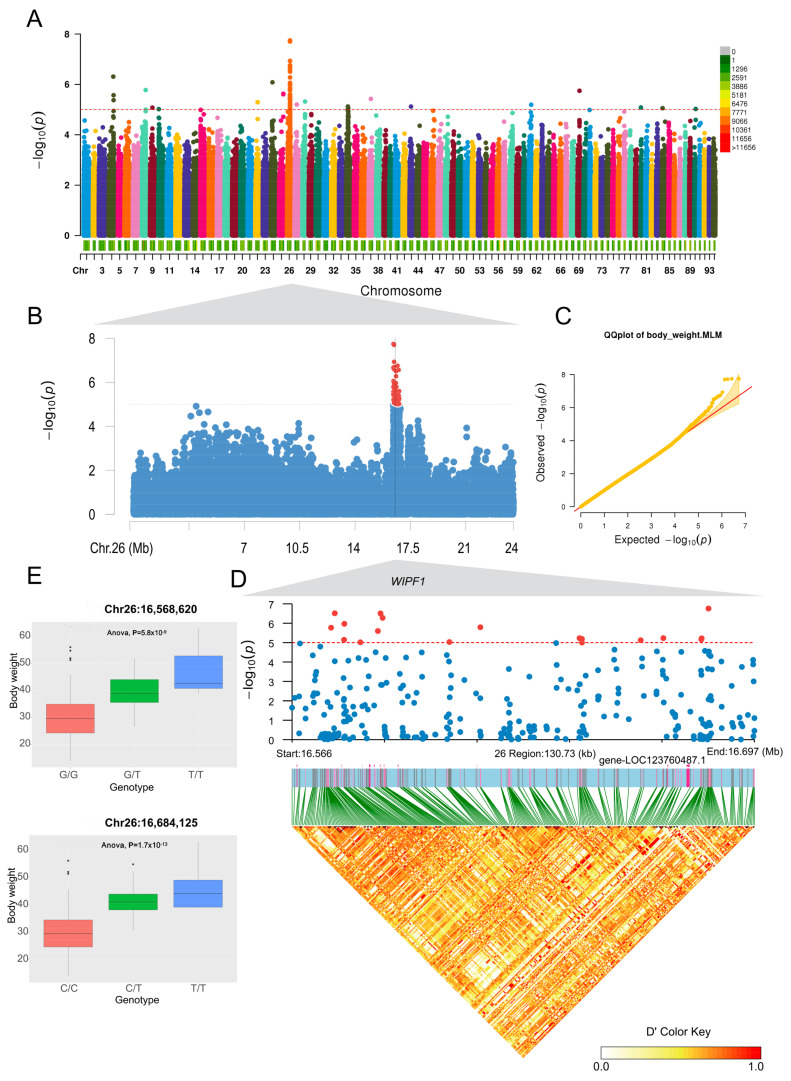
The results of genome-wide association study for body weight. (**A**) Manhattan plots of the whole genome-wide association study; (**B**) local Manhattan plots for chromosome 26; (**C**) QQ-plots of the whole genome-wide association study; (**D**) local Manhattan plots and linkage disequilibrium heatmap of gene LOC123760487.1 (*WIPF1*); (**E**) box plots of loci Chr26:16,568,620 and Chr26:16,684,125. Horizontal red dashed lines indicate the whole-genome significance threshold (*p*-value = 1 × 10^−5^).

To further screen for genetic markers associated with superior traits in the red swamp crayfish, heritability analysis showed relatively high heritability for body weight, body weight/body length, and body length/abdominal weight (Figure S1). Genome-wide association study showed that 52 SNPs were identified to be significantly associated with body weight (*p*-value < 1 × 10^−5^). Twenty-eight of these loci were clustered on chromosome 26 (Figure 5A–C). According to the gene structure, these 28 loci were concentrated on *WIPF1* on chromosome 26, and all of them were located on an intron of *WIPF1* (Figure 5D). Further, we found that SNPs on WIPF1 showed strong correlations with body weight, e.g., for loci Chr26:16,568,620 and Chr26:16,684,125, the mutant genotype ‘T’ was significantly positively correlated with body weight (*p* ≤ 5.8 × 10^−9^), and was significantly correlated with the corresponding pure haplotypes which increased the average body weight compared to the corresponding haplotypes (Figure 5E). The results indicated that *WIPF1* may be a body weight-related candidate gene.

### 3.5. Expression of WIPF1 in Shrimp Fry of the Same Day Old Size Difference Population

**Figure 6:**
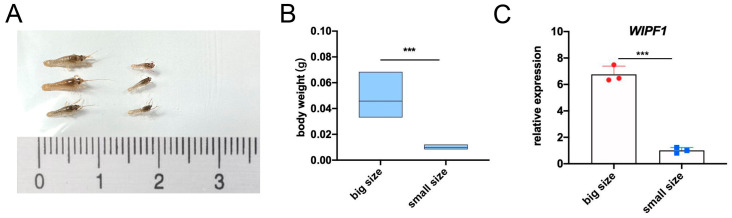
The red swamp crayfish larvae observations and *WIPF1* expression levels in small and large populations of the red swamp crayfish larvae. (**A**) Same-day-old larvae observations; (**B**) Same-day-old larvae body weight statistics; (**C**) *WIPF1* expression levels in small and large populations of growth-differentiated larvae (*** *p* < 0.001).

There were body size differences between crayfish larvae reared in the same batch and we examined the expression of *WIPF1* in growth-differentiated populations and found that the expression level of *WIPF1* was significantly higher in large populations (body weights ranging from 0.0331 g to 0.0684 g) than that of small populations (0.0084 g to 0.0121 g), further suggesting that *WIPF1* is a functional gene related to body weight (Figure 6A–C).

## 4. Discussion

In this study, we constructed three different red swamp crayfish lineage selection populations and performed whole-genome resequencing on the constructed lineage selection populations and wild red swamp crayfish populations from four regions (Nanx, Mil, Caish, and Wangc) in Hunan Province. The phylogenetic tree was constructed by whole genome sequencing analysis and found that the phylogenetic tree was clustered according to geographic regions, or each family line was clustered separately. Principal component analysis showed that there was a clear separation between the family group and the wild group, and that JS03 and JS08 were clustered separately. Ancestral composition analyses showed that most lineages appeared to be more homogeneous at K = 5, reflecting the differentiation of several populations from others in the lineage generations. Linkage disequilibrium results showed that wild populations decayed slightly faster than familial populations. Gene flow analyses showed localized gene flow between familial populations, whereas there was no gene flow between Nanx and Wangc populations and familial populations. Further, by the genome-wide association study, 52 SNPs were identified that were significantly associated with body weight traits, of which 28 loci were clustered on the intron of *WIPFI* on chromosome 26, for loci Chr26:16,568,620 and Chr26:16,684,125; the alternative allele ‘T’ was significantly positively correlated with body weight and was significantly correlated with the corresponding pure haplotypes which increased the average body weight compared to the corresponding haplotypes. Further, it was found that the expression level of *WIPF1* in the large group was significantly higher than that in the small group, and the candidate genes for growth were screened out. The results of the study provide a theoretical reference for exploring the growth mechanism of crayfish, as well as a reliable theoretical guidance basis for the in-depth genetic improvement of red swamp crayfish.

Genomic data were generated from a large number of family line crayfish and wild crayfish to characterize the gradual infiltration of adaptive genes and genetic changes during crayfish selection. In this study, we performed genome resequencing with a 6-fold coverage depth on 197 crayfish, which came from the familial line selection populations of our research team and wild populations in four regions of Hunan Province. Neighbor-joining tree (NJ) analysis showed that the family lineages could be divided into separate clusters, such as JS02, JS03, JS08, and JS42, the genetic distance from the natural population is relatively far. Under family deviation selection, the deviation of each lineage from its family mean was determined in absolute units, and the lineage with the largest deviation was selected from the totality of the family lineages [35]. PCA analyses further supported this discrepancy, i.e., there was a tendency for JS03 and JS08 to form distinctive new germplasm resources [36]. Ancestral component analysis showed that most of the family lineage groups, including JS03 and JS08, had the same ancestral origin, suggesting that there was no mixing between the families during the selection breeding process. This is likely related to our pairing strategy of selection within the family lineage [37]. From the linkage disequilibrium results, the wild populations decayed slightly faster than the familial populations, which may be due to genetic drift and different genomic structures [38]. The rate of LD decay tends to vary considerably between species or between subpopulations of the same species. In the same population strand, LD decay is slow, indicating selection on the population [31,39]. The results of the gene flow analysis showed that there was localized gene flow between the family lines, no gene flow between the Nanx and Wangc groups with the family lines, and gene flow between the Mil and Caish with the family lines. On the one hand, it may be that these gene exchange groups of individuals share common alleles and are more likely to occur between groups of closer kinship [40]. On the other hand, Caish and Mil are adjacent and connected to the East Dongting Lake, and both with family groups originating from the East Dongting Lake system, while the Nanx and Wangc groups originated from other artificially cultured closed water bodies. Our results provide further evidence of migratory events between all family samples and wild-type samples.

Currently, the utilization of crustacean genomics still lags far behind those in other animals, but the rapid selection of new varieties requires the use of genomic information [17]. Through genome-wide association analysis, we identified 52 weight-related SNPs, of which 28 sites were clustered on WIPF1 on chromosome 26, and all of them were located on an intron of WIPF1. Intron SNPs may affect gene expression by affecting splicing, mRNA stability, or transcription factor regulation [9,41]. The SNPs on WIPF1 showed a strong correlation with body weight, suggesting that WIPF1 may be a candidate gene related to body weight. The *WIPF1* gene encodes WASP-interacting protein family member 1, also known as *WIPF1*. It is an important cytoskeletal protein involved in the organization and polymerization of the actin cytoskeleton and is associated with cell proliferation and invasion [12,25]. It has been reported to have a regulatory effect on the PI3K/AKT signaling pathway [42,43,44,45]. In mammalian germ cell development, the *WIPF1* gene interacts with Nemo-like kinase (NLK), leading to an enhancement of the Wnt signaling pathway and promoting sperm development and maturation [46]. It has also been shown that WIPF1 has oncogene characteristics and plays an important role in promoting tumor growth and metastasis [24]. Currently, there is no report of the *WIPF1* gene in carapace; in our study, we found that the expression level of *WIPF1* was significantly higher in the large population of juvenile shrimp of the same age than in the small population, further suggesting *WIPF1* as a body weight-related candidate gene.

## 5. Conclusions

In conclusion, this study successfully established a full-sib breeding population of *P. clarkii* and generated 13 family populations in the F3 generation. After three years of breeding, we observed that the family populations exhibited higher genetic purity, slower decay rates, and more stable candidate strains such as JS03, JS08, and JS42 were obtained, which provided a material platform for the creation of new varieties of *P. clarkii*. Furthermore, genome-wide association analysis identified 52 significant SNP loci associated with body weight, with 28 loci concentrated in the intron of the *WIPF1* gene on chromosome 26. This is the first report of a gene locus linked to body weight in *P. clarkii*. The strong correlation between SNP variation in WIPF1 and body weight, along with differential gene expression in distinct size groups, highlights *WIPF1* as a key candidate gene influencing body weight in *P. clarkii*. These results provide a theoretical foundation for molecular marker-assisted breeding and genetic improvement of crayfish.

## Figures and Tables

**Table 1:** Population genetic diversity index.

Pop	SNP Number	SNP Density (SNP/Kb)	Expected Heterozygosity (He)	Observed Heterozygosity (Ho)	Shannon’s Diversity Index	Minor Allele Frequency (MAF)	Polymorphism Information Content (PIC)	Nucleotide Diversity (π)	Inbreeding Coefficient (F)
JS02	8,627,048	3.15	0.2031 ± 0.2050	0.2171 ± 0.2687	0.3009 ± 0.2932	0.1558 ± 0.1730	0.2119 ± 0.2357	0.0011 ± 0.0014	0.0183 ± 0.0620
JS03	8,627,048	3.15	0.2286 ± 0.1983	0.2200 ± 0.2355	0.3426 ± 0.2798	0.1724 ± 0.1684	0.2100 ± 0.1932	0.0011 ± 0.0013	0.0574 ± 0.0812
JS05	8,627,048	3.15	0.2135 ± 0.2056	0.2328 ± 0.2745	0.3159 ± 0.2931	0.1643 ± 0.1749	0.2241 ± 0.2392	0.0011 ± 0.0014	0.0002 ± 0.0914
JS08	8,627,048	3.15	0.2652 ± 0.1762	0.2362 ± 0.2053	0.4048 ± 0.2390	0.1944 ± 0.1549	0.2334 ± 0.1590	0.0012 ± 0.0013	0.1173 ± 0.0905
JS19	8,627,048	3.15	0.2045 ± 0.2059	0.2140 ± 0.2664	0.3026 ± 0.2941	0.1574 ± 0.1743	0.2111 ± 0.2339	0.0011 ± 0.0014	0.0398 ± 0.0703
JS24	8,627,048	3.15	0.2146 ± 0.2048	0.2343 ± 0.2732	0.3180 ± 0.2921	0.1646 ± 0.1738	0.2247 ± 0.2374	0.0011 ± 0.0014	−0.0009 ± 0.0763
JS42	8,627,048	3.15	0.2253 ± 0.2028	0.2677 ± 0.2945	0.3342 ± 0.2890	0.1727 ± 0.1738	0.2534 ± 0.2595	0.0012 ± 0.0014	−0.0667 ± 0.1043
FX05	8,627,048	3.15	0.2105 ± 0.2027	0.2355 ± 0.2771	0.3130 ± 0.2897	0.1602 ± 0.1712	0.2250 ± 0.2410	0.0011 ± 0.0014	−0.0257 ± 0.0627
FX10	8,627,048	3.15	0.1995 ± 0.2056	0.2210 ± 0.2864	0.2947 ± 0.2949	0.1542 ± 0.1752	0.2228 ± 0.2583	0.0011 ± 0.0014	0.0341 ± 0.0091
FX19	8,627,048	3.15	0.2405 ± 0.1936	0.2362 ± 0.2514	0.3607 ± 0.2737	0.1808 ± 0.1669	0.2416 ± 0.2160	0.0012 ± 0.0014	0.0983 ± 0.1386
FX21	8,627,048	3.15	0.2047 ± 0.2043	0.2124 ± 0.2637	0.3037 ± 0.2917	0.1569 ± 0.1730	0.2107 ± 0.2306	0.0011 ± 0.0013	0.0498 ± 0.0748
FX22	8,627,048	3.15	0.2340 ± 0.1982	0.2437 ± 0.2650	0.3492 ± 0.2813	0.1771 ± −0.1694	0.2412 ± 0.2278	0.0012 ± 0.0014	0.0457 ± 0.1416
RA08	8,627,048	3.15	0.2051 ± 0.2088	0.2558 ± 0.3216	0.3010 ± 0.2997	0.1615 ± 0.1812	0.2544 ± 0.2978	0.0012 ± 0.0015	−0.0273 ± 0.0679
Mil	8,627,048	3.15	0.3128 ± 0.1462	0.2636 ± 0.1835	0.4764 ± 0.1842	0.2280 ± 0.1401	0.2761 ± 0.1336	0.0014 ± 0.0014	0.1747 ± 0.0590
Caish	8,627,048	3.15	0.3185 ± 0.1421	0.2761 ± 0.1826	0.4843 ± 0.1773	0.2323 ± 0.1382	0.2818 ± 0.1309	0.0015 ± 0.0014	0.1528 ± 0.0773
Nanx	8,627,048	3.15	0.3071 ± 0.1502	0.2472 ± 0.1847	0.4682 ± 0.1909	0.2238 ± 0.1422	0.2705 ± 0.1370	0.0014 ± 0.0014	0.2115 ± 0.0907
Wangc	8,627,048	3.15	0.3159 ± 0.1450	0.2594 ± 0.1790	0.4803 ± 0.1826	0.2304 ± 0.1392	0.2766 ± 0.1289	0.0014 ± 0.0014	0.2033 ± 0.1526
all	8,627,048	3.15	0.3190 ± 0.1324	0.2431 ± 0.1612	0.4884 ± 0.1580	0.2302 ± 0.1330	0.2673 ± 0.1029	0.0014 ± 0.0014	0.2365 ± 0.1013

## Data Availability

The original contributions presented in the study are included in the article and Supplementary Material, further inquiries can be directed to the corresponding authors.

## Supplementary Materials

The following supporting information can be downloaded at: https://www.mdpi.com/article/10.3390/genes16020174/s1, Figure S1. Analysis of the heritability of the family crayfish and the wild crayfish; Figure S2. LD linkage disequilibrium analysis of different families and wild populations; Table S1. Primer sequences for qPCR analysis; Table S2. Phenotypic indicators in 117 family samples and 80 wild samples. Table S3. Sequencing output data statistics and comparative efficiency.
