# Supplementary material for: Construction of the Red Swamp Crayfish (Procambarus clarkii) Family Selection Population and Whole Genome Sequencing to Screen WIPFI Candidate Genes Related to Growth
Source: Genes (Basel). 2025 Jan 31;16(2):174. doi: 10.3390/genes16020174 (PMC11855636; doi:10.3390/genes16020174)
Supplement: Supplementary file 1 [file genes-16-00174-s001.zip › Supplementary Material-Figure S1-S2_Table S-S2 .pdf]

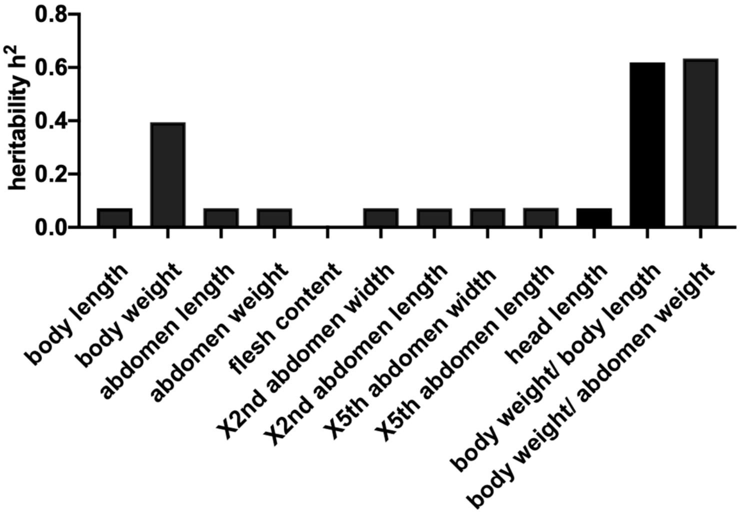

Figure S1. Analysis of the heritability of the family crayfish and the wild crayfish.

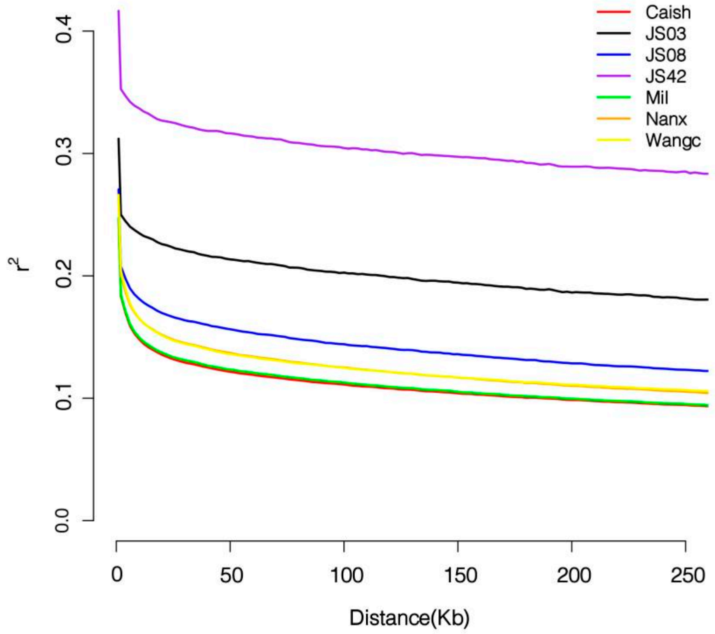

Figure S2. LD linkage disequilibrium analysis of different families and wild populations.

Table S1. Primer sequences for qPCR analysis

Gene name	Forward Primer (5'-3')	Reverse Primer (5'-3')	product length
β-actin	F: AAGGATCTGTACGCCAACAC	R: GAACCGCCAATCCATACAGAG	161bp
WIPF1	F: AGCCCTTCCAAGTAAGCCAGT	R: ATTTGGCTGTAGTCCCGACCC	124bp

Table S2. Phenotypic indicators in 117 family samples and 80 wild samples.

Sample Sources	body length (cm)	body weight (g)	abdomen length (cm)	abdomen weight (g)	flesh content%	X2nd abdomen width (cm)	X2nd abdomen length (cm)
JS02	93.65±8.27^{ab}	31.06±5.05^{abc}	34.36±2.85^{abc}	5.95±0.51^{ab}	8.99±1.94^{a}	16.44±1.45^{ab}	6.35±0.52^{ab}
JS03	90.77±7.75^{ab}	26.75±5.23^{ab}	33.37±2.66^{abc}	5.77±0.47^{ab}	10.38±1.54^{ab}	15.93±1.36^{ab}	6.17±0.49^{ab}
JS05	98.54±4.20^{ab}	34.80±5.42^{bc}	36.04±1.44^{abc}	6.25±0.25^{ab}	9.54±1.25^{a}	17.30±0.73^{ab}	6.66±0.26^{ab}
JS08	95.73±7.96^{ab}	29.99±7.93^{abc}	35.08±2.74^{abc}	6.08±0.49^{ab}	10.99±2.59^{ab}	16.80±1.39^{ab}	6.48±0.50^{ab}
JS19	88.38±3.18^{ab}	25.30±4.39^{ab}	32.55±1.09^{abc}	5.62±0.19^{ab}	10.31±1.29^{ab}	15.52±0.55^{ab}	6.02±0.20^{ab}
JS24	86.30±6.48^{a}	22.38±4.66^{a}	31.83±2.22^{a}	5.49±0.40^{ab}	10.94±0.60^{ab}	15.15±1.13^{a}	5.89±0.41^{a}
JS42	92.18±6.80^{ab}	29.00±7.80^{abc}	33.86±2.34^{abc}	5.86±0.42^{ab}	9.77±1.44^{a}	16.18±1.19^{ab}	6.26±0.43^{ab}
FX05	87.91±7.44^{ab}	26.20±2.59^{ab}	32.39±2.56^{ab}	5.59±0.46^{ab}	9.67±1.60^{a}	15.43±1.30^{ab}	5.99±0.47^{ab}
FX10	91.65±6.27^{ab}	27.07±3.27^{ab}	33.68±2.15^{abc}	5.82±0.38^{ab}	10.26±0.96^{ab}	16.09±1.10^{ab}	6.23±0.39^{ab}
FX19	99.21±9.84^{ab}	35.61±9.04^{bc}	36.27±3.38^{abc}	6.29±0.60^{b}	9.89±1.71^{a}	17.41±1.73^{ab}	6.71±0.62^{ab}
FX21	91.38±+7.54^{ab}	25.76±5.01^{ab}	33.58±2.59^{abc}	5.81±0.46^{ab}	11.14±2.14^{ab}	16.04±1.32^{ab}	6.21±0.47^{ab}
FX22	93.07±4.97^{ab}	34.43±1.71^{bc}	34.16±1.71^{abc}	5.91±0.30^{ab}	8.20±1.43^{a}	16.34±0.87^{ab}	6.32±0.31^{ab}
RA08	101.15±9.92^{b}	34.36±12.47^{bc}	36.94±3.41^{bc}	6.41±0.61^{b}	11.69±3.82^{ab}	17.75±1.74^{b}	6.83±0.63^{ab}
Mil	99.00±6.17^{ab}	39.81±9.19^{c}	36.20±2.12^{abc}	6.28±0.38^{ab}	9.08±1.26^{a}	17.38±1.08^{ab}	6.69±0.39^{ab}
Caish	101.69±7.97^{b}	39.65±6.56^{c}	37.13±2.74^{c}	6.45±0.49^{b}	9.56±2.12^{a}	17.85±1.40^{b}	6.87±0.50^{b}
Nanx	90.20±9.11^{ab}	31.32±6.95^{abc}	33.17±3.13^{abc}	5.73±0.56^{ab}	8.68±1.99^{a}	15.83±1.60^{ab}	6.13±0.57^{ab}
Wangc	89.86±6.58^{ab}	21.22±4.71^{a}	33.98±1.91^{abc}	5.31±0.93^{a}	13.50±2.73^{b}	15.76±1.46^{ab}	6.34±0.59^{ab}

[1] Mil, MiLuo; Caish, CaiSangHu; Nanx, NanXian; Wangc, WangCheng.

[2]Different superscript letters in the same row indicate significant differences ($P < 0.05$).
